# *Contextual factors influencing the performance of clinical preceptors: A scoping review protocol*

**DOI:** 10.1016/j.mex.2026.104131

**Published:** 2026-08-29

**Authors:** Bárbara Porter Jalife, Andrea Rodríguez Figueroa, Ivonne Riquelme Espinoza, María José Montecinos Mundaca, Sigrid Dömke Schneider

**Affiliations:** a School of Midwifery and Obstetrics, Faculty of Health Care, Universidad San Sebastián, Concepción, 4030000, Chile; b School of Midwifery and Obstetrics, Faculty of Health Care, Universidad San Sebastián, Santiago, 8320000, Chile; c School of Midwifery and Obstetrics, Faculty of Health Care, Universidad San Sebastián, Valdivia, 5090000, Chile; d School of Midwifery and Obstetrics, Faculty of Health Care, Universidad San Sebastián, Puerto Montt, 5480000, Chile

**Keywords:** Preceptor, Clinical supervisor, Contextual factors, Organizational aspects, Health professions, Clinical teaching, Preceptorship

## Abstract

The clinical preceptor plays a fundamental role in health professionals training. Their performance influences the acquisition of clinical competencies, the development of professional identity and the integration of ethical standards by students. Although interventions exist to strengthen preceptors' teaching competencies, there is a gap concerning the contextual factors that affect the transfer of these competencies into real clinical training settings. The aim of the present study is to describe contextual factors that influence the performance of clinical preceptors in health professions, explicitly documenting differences across health disciplines and by countries’ income levels. The review will follow JBI and PRISMA-ScR guidelines.

## Highlights

• Identification of contextual factors that affect clinical preceptor performance.
• Comparison of differences across health disciplines and by the income level of the countries included in the study.
• Synthesis of evidence based on JBI and PRISMA-ScR methodological guidance.

## Graphical abstract

**Figure:**
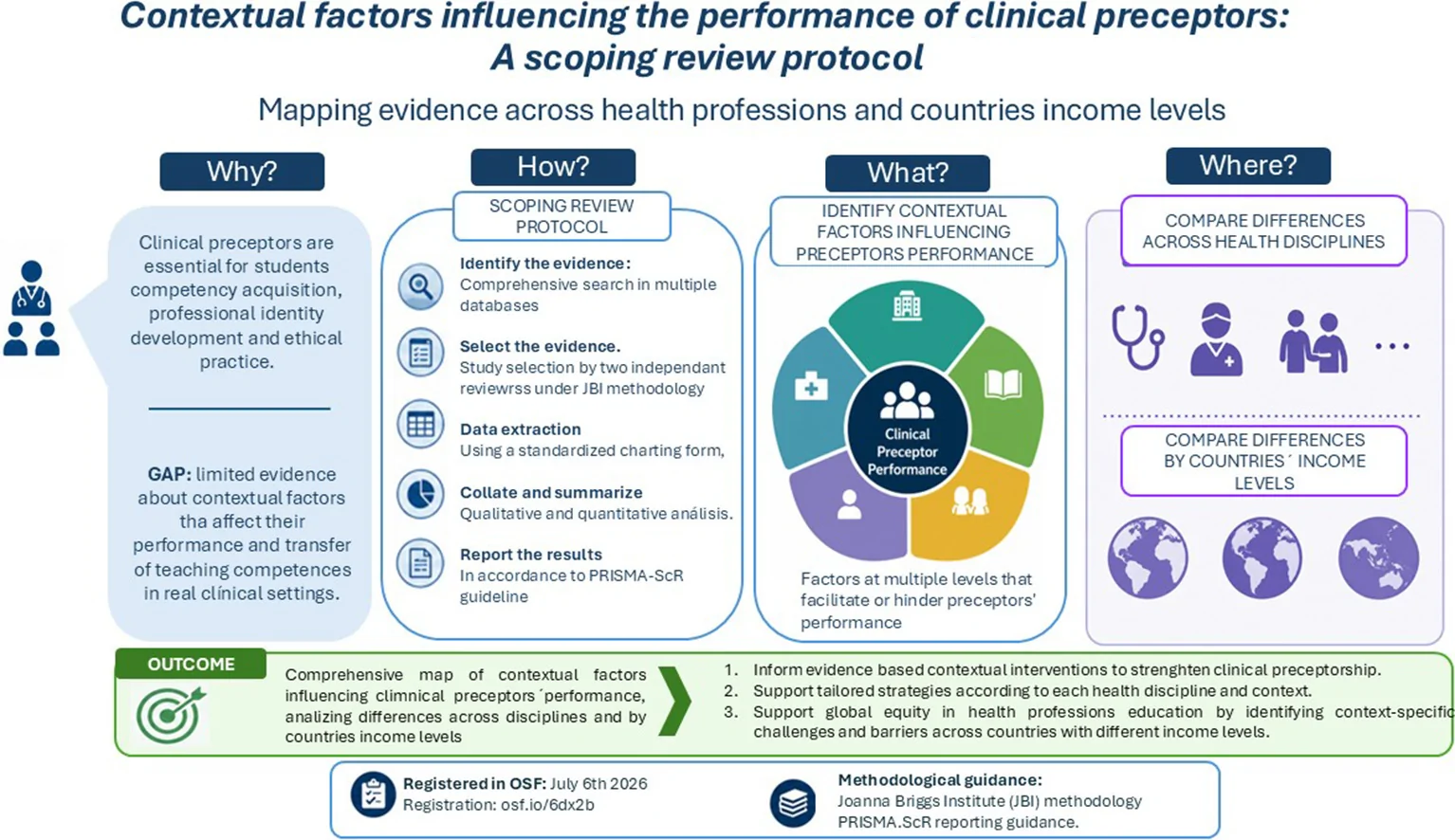

## Specifications table

**Table:** 

**Subject area**	Medicine and Dentistry
**More specific subject area**	*Health professions*
**Name of your protocol**	Contextual factors influencing the performance of clinical preceptors: A scoping review protocol.
**Reagents/tools**	*None.*
**Experimental design**	*Not applicable.*
**Trial registration**	*Not applicable.*
**Ethics**	*This scoping review will use data form previous published articles and will not involve human or animal participants. Therefore, ethical approval and informed consent are not required for this research.*
**Value of the Protocol**	• *This scoping review will systematize the existing literature on contextual factors that influence clinical preceptors' performance in their role training health professionals.* • *To ensure rigor, transparency, and reproducibility, this review is grounded in the principles described by JBI and PRISMA.* • *The findings will enable an understanding of the clinical, organizational, institutional, and social factors that affect preceptor performance within the context of clinical training for health professionals, along with potential differences across health professions and between countries according to income level.*

## Background

The clinical preceptor role in health professions constitutes a central axis linking academic training and professional practice [1]. The preceptor functions as a role model, supervisor, facilitator of learning experiences, and agent of socialization toward competent professional practice [2,3]. Accordingly, their behavior decisively influences the acquisition of critical competencies, the construction of professional identity, and the internalization of ethical standards by students [4].

Essential competencies for the clinical preceptor role in health programs have been described. According to Bartlett [5], there are 17 core competencies required for effective preceptorship, including specialized professional knowledge, clinical and communication skills, supervisory and teaching abilities, the capacity to provide effective feedback, and a range of formative attributes and attitudes that support mentorship and the learner’s progressive development. There is also evidence of interventions aimed at developing these skills through workshops, online courses, simulation strategies, and structured pedagogical models [[6], [7], [8], [9], [10]]. These training initiatives are heterogeneous, focusing on different skill sets and varying in learning strategies, duration, frequency, and evaluation methods [11]. Although evidence suggests these educational activities have a positive effect, the literature reports several extrinsic or contextual factors that may hinder the transfer and application of those competencies into everyday clinical training contexts [12].

Several authors propose that extrinsic or contextual factors influence preceptors' practice [[13], [14], [15]]. Nordi [13] defines these as elements related to the environment in which preceptorship occurs and to the institutional and employment conditions that shape its practice; these factors would affect both the quality and the sustainability of the preceptor role. These factors can be grouped into three domains: (1) university–preceptor relationship, including recognition, incentives, institutional orientation, and the perceived value of the role; (2) conditions of role practice, such as workload and clinical duties, time pressure, unpredictability of clinical situations, bureaucracy, service productivity demands, limited time for teaching, and infrastructure problems; and (3) recruitment and retention, encompassing remuneration, time allocation, teaching incentives, hierarchical dependence, continuing professional development, and internal and external competition to attract preceptors [15]. At the macro level, national regulations and a country’s income level may affect preceptor performance; for example, lower income levels have been associated with an increased risk of burnout among these professionals [16,17].

Previous reviews [5,6,11,18,19] have addressed this topic across various professions, including medicine, nursing, and pharmacy. These reviews emphasize the description of the competencies required and the educational strategies for performing the preceptor role. However, they do not provide a systematic synthesis of the contextual factors that may influence the transfer and application of those competencies in clinical training settings, such as clinical-environment factors, organizational or institutional factors, including existing regulations or the income level of the countries.

The available literature is fragmented and often focuses solely on individual skills, leaving a gap in the understanding and systematization of the contextual factors that may influence the successful transfer of those competencies to the training environment.

## Description of protocol

### Design and research questions

Due to the context and the identified gap, a scoping review is an appropriate method to map and broadly systematize the existing evidence on contextual factors that influence the transfer of those competencies [20].

The objective of the present scoping review is to describe the contextual factors (clinical, organizational, institutional or other) that influence the performance and the transfer of preceptors’ competencies into the clinical training environment.

The primary research question is framed using the Population–Concept–Context (PCC) framework. In this case, the population comprises preceptors/tutors/clinical supervisors; the concept is contextual factors; and the context is preceptorship/clinical supervision as a higher education training activity within health professions. The guiding question for this review is: What does the literature describe about the contextual factors that affect preceptors’ performance in the context of clinical training for health professionals?

To ensure a detailed understanding of the phenomenon, secondary questions were formulated that relate to the primary question. These questions will allow deeper exploration of the characteristics and nature of the contextual factors, including differences that may exist across health disciplines and between countries with different income levels. The secondary questions are:

(1) At what levels can contextual factors that affect preceptors’ performance in clinical health-professional training be classified?
(2) What categories of contextual factors emerge from literature as influencing preceptors’ effectiveness in clinical supervision?
(3) How do contextual factors and their impact on preceptor performance differ across health professions?
(4) How do contextual factors and preceptor performance vary according to country income level?

To ensure transparency and methodological rigor, this scoping review was registered on July 6, 2026, in the Open Science Framework (OSF) under the registration number osf.io/6dx2b. The review will be conducted in accordance with the methodological guidance provided by the JBI and will adhere to the reporting recommendations of the PRISMA Extension for Scoping Reviews (PRISMA-ScR) [21].

### Elegibility criteria

The inclusion and exclusion criteria were formulated based on the PCC model, as detailed below:

#### P – participants

**Inclusion:** We will include literature that considers clinical preceptors, clinical supervisors, or clinical tutors as the population, when acting in their formative role with undergraduate or postgraduate students in health professions.

**Exclusion:** Studies focusing on clinical preceptors in veterinary medicine, animal health, or other non-human health professions will be excluded.

#### C- concept:

**Inclusion:** Studies that explicitly examine contextual, organizational, structural, relational, or regulatory factors that shape the clinical environment and influence preceptor performance will be included.

**Exclusion:** Studies focusing exclusively on individual preceptor characteristics (e.g., personality, intrinsic motivation, or cognitive styles), clinical or pedagogical competencies without consideration of contextual factors, evaluations of educational programs that do not examine contextual influences, or educational interventions described solely in curricular or methodological terms will be excluded.

#### C- context

**Inclusion:** Studies conducted in the context of clinical education within healthcare settings, including clinical internships, professional placements, and authentic healthcare environments (e.g., hospitals, primary care settings, clinics, and community health centers) in higher education will be included.

**Exclusion:** Studies conducted in clinical simulation settings without interaction with real patients or in virtual education environments without in-person clinical practice will be excluded.

### Type of sources

Eligible sources for inclusion will encompass all types of publications, regardless of methodological approach. Studies employing experimental, quasi-experimental, observational, cohort, and case–control designs will be eligible. Qualitative, mixed-methods, systematic review, scoping review, and meta-analysis studies will also be considered.

Publications in both indexed and non-indexed peer-reviewed journals will be included, as well as grey literature, including master's theses and undergraduate dissertations available in institutional repositories, doctoral dissertations, and conference proceedings. No publication date restrictions will be applied to ensure the broadest possible coverage of the relevant literature.

Publications in English, Spanish, and Portuguese will be eligible, provided that the full text is accessible. If the full text is not readily available, the corresponding authors will be contacted to request access. Studies for which the full text cannot be obtained within a reasonable timeframe will be excluded from the review.

The inclusion of diverse types of evidence sources is essential to comprehensively capture the existing evidence on the phenomenon under investigation and to answer the research question with the necessary depth and breadth.

### Search strategy

Potentially eligible articles will be identified through a systematic search of the following electronic databases: Web of Science (WoS), Scopus, PubMed, ERIC, and Google Scholar.

The search strategy was developed by identifying relevant terms from the Medical Subject Headings (MeSH) thesaurus and complementing these with keywords extracted from relevant articles, as well as free-text terms, to maximize both the sensitivity and specificity of the search. The aim is to retrieve the largest possible number of relevant studies while minimizing the inclusion of records that are not pertinent to the research question. The search strategy was developed according to the Population–Concept–Context (PCC) framework recommended for Scoping Reviews. The initial search strategy was constructed using Web of Science Query Builder. The strategy was then adapted to the specific syntax, field codes and search functionalities of Scopus and PubMed. Boolean operators (OR/AND), truncation, and database-specific indexing terms were used as appropriate (Table 1). This strategy will subsequently be adapted for the other databases included in the review (ERIC, Google Scholar).

**Table 1:** Search strategy.

Database	Search Strategy
WoS	(((TS=(preceptor* OR clinical preceptor* OR nurse preceptor* OR clinical facilitator* OR clinical educator* OR clinical teacher* OR clinical instructor* OR clinical supervisor* OR practice educator* OR practice teacher* OR clinical mentor* Or clinical tutor* OR clinical coach* OR student supervisor* OR clinical learning facilitator* OR clinical accompaniment)) AND TS=(performance OR work performance OR job performance OR professional performance OR role performance OR teaching performance OR preceptor effectiveness OR clinical educator effectiveness OR role effectiveness OR work functioning OR professional functioning OR teaching competence OR supervisory competence OR role fulfillment OR performance at work)) AND TS=(Organizational aspects OR Learning Environment OR Work Environment OR Organizational Climate OR organizational culture OR workload OR work overload Or work demands Or time pressure Or time constraint OR lack of time OR work schedule* OR working hours OR overtime OR role conflict OR role strain OR role ambiguity OR role overload OR dual role OR patient load OR patient volume OR number of students OR student caseload OR student-to-preceptor ratio OR documentation OR paperwork OR administrative burden OR staff turnover OR understaffing OR staff shortage OR organizational support OR managerial support OR supervisor support OR peer support OR resources OR teamwork OR communication OR leadership OR professional development OR preceptor training OR Extrinsic aspects OR Contextual aspects OR Cultural Context OR Social Environment OR health polic* OR healthcare polic* OR health workforce polic* OR clinical education polic* OR education polic* OR public health polic* OR health governance OR health system governance OR professional regulation OR regulatory framework OR licensure accreditation OR professional standard* OR clinical education standard* OR preceptorship standard* OR legal requirement* OR regulatory requirement* OR healthcare funding OR clinical education funding OR preceptor funding OR financial incentive* OR payment for precepting OR health workforce OR health workforce shortage* OR nursing shortage* OR preceptor shortage* OR clinical education infrastructure)) AND TS=(Preceptorship* OR clinical education OR clinical teaching OR clinical supervision OR clinical facilitation OR clinical placement* OR student placement* OR practicum OR internship OR health professions education OR nursing education OR medical education OR health care profession* OR medical residenc* OR hospital* OR health service* OR primary care OR healthcare organization*)
**Scopus**	(TITLE-ABS-KEY ("preceptor*" OR "clinical preceptor*" OR "nurse preceptor*" OR "clinical facilitator*" OR "clinical educator*" OR "clinical teacher*" OR "clinical instructor*" OR "clinical supervisor*" OR "practice educator*" OR "practice teacher*" OR "clinical mentor*" OR "clinical tutor*" OR "clinical coach*" OR "student supervisor*" OR "clinical learning facilitator*" OR "clinical accompaniment") AND TITLE-ABS-KEY ("performance" OR "job performance" OR "professional performance" OR "role performance" OR "teaching performance" OR "preceptor effectiveness" OR "clinical educator effectiveness" OR "role effectiveness" OR "work functioning" OR "professional functioning" OR "teaching competence" OR "supervisory competence" OR "role fulfillment" OR "performance at work") AND TITLE-ABS-KEY ("organizational aspect*" OR "organisational aspect*" OR "learning environment" OR "work environment" OR "organizational climate" OR "organisational climate" OR "organizational culture" OR "organisational culture" OR "workload" OR "work overload" OR "work demand*" OR "time pressure" OR "time constraint*" OR "lack of time" OR "work schedule*" OR "working hours" OR overtime OR "role conflict" OR "role strain" OR "role ambiguity" OR "role overload" OR "dual role" OR "patient load" OR "patient volume" OR "number of students" OR "student caseload" OR "student-to-preceptor ratio" OR documentation OR paperwork OR "administrative burden" OR "staff turnover" OR understaffing OR "staff shortage" OR "organizational support" OR "organisational support" OR "managerial support" OR "supervisor support" OR "peer support" OR resource* OR teamwork OR communication OR leadership OR "professional development" OR "preceptor training" OR "extrinsic aspect*" OR "contextual aspect*" OR "cultural context" OR "social environment" OR "health polic*" OR "healthcare polic*" OR "health workforce polic*" OR "clinical education polic*" OR "education polic*" OR "public health polic*" OR "health governance" OR "health system governance" OR "professional regulation" OR "regulatory framework*" OR licensure OR accreditation OR "professional standard*" OR "clinical education standard*" OR "preceptorship standard*" OR "legal requirement*" OR "regulatory requirement*" OR "healthcare funding" OR "clinical education funding" OR "preceptor funding" OR "financial incentive*" OR "payment for precepting" OR "health workforce" OR "health workforce shortage*" OR "nursing shortage*" OR "preceptor shortage*" OR "clinical education infrastructure") AND TITLE-ABS-KEY ("preceptor*" OR "clinical education" OR "clinical teaching" OR "clinical supervision" OR "clinical facilitation" OR "clinical placement*" OR "student placement*" OR "practicum" OR "internship" OR "health professions education" OR "nursing education" OR "medical education" OR "health care profession*" OR "healthcare profession*" OR "medical residenc*" OR hospital* OR "health service*" OR "primary care" OR "healthcare organization*" OR "healthcare organisation*"))
**PubMed**	((((preceptor*[tiab] OR "clinical facilitator*"[tiab] OR "clinical educator*"[tiab] OR "clinical teacher*"[tiab] OR "clinical instructor*"[tiab] OR "clinical supervisor*"[tiab] OR "practice educator*"[tiab] OR "clinical mentor*"[tiab] OR "student supervisor*"[tiab] OR "clinical learning facilitator*"[tiab] OR "clinical accompaniment"[tiab] OR "Preceptorship"[Mesh])) AND (("performance"[tiab] OR "work performance"[tiab] OR "job performance"[tiab] OR "professional performance"[tiab] OR "role performance"[tiab] OR "teaching performance"[tiab] OR "preceptor effectiveness"[tiab] OR "work functioning"[tiab] OR "teaching competence"[tiab] OR "supervisory competence"[tiab] OR "Professional Competence"[Mesh] OR "Teaching"[Mesh]))) AND ((workload[tiab] OR "work overload"[tiab] OR "work demand*"[tiab] OR "time pressure"[tiab] OR "time constraint*"[tiab] OR "lack of time"[tiab] OR "role conflict"[tiab] OR "role strain"[tiab] OR "role ambiguity"[tiab] OR "role overload"[tiab] OR "dual role"[tiab] OR "patient load"[tiab] OR "patient volume"[tiab] OR "student caseload"[tiab] OR documentation[tiab] OR paperwork[tiab] OR "administrative burden"[tiab] OR "staff turnover"[tiab] OR understaffing[tiab] OR "staff shortage"[tiab] OR "organizational support"[tiab] OR "organisational support"[tiab] OR "supervisor support"[tiab] OR "work environment"[tiab] OR "organizational culture"[tiab] OR "organisational culture"[tiab] OR "health polic*"[tiab] OR "health workforce polic*"[tiab] OR "clinical education polic*"[tiab] OR "professional regulation"[tiab] OR "regulatory framework*"[tiab] OR accreditation[tiab] OR licensure[tiab] OR funding[tiab] OR "health workforce shortage*"[tiab] OR "preceptor shortage*"[tiab] OR "Health Policy"[Mesh] OR "Health Workforce"[Mesh] OR "Workplace"[Mesh] OR "Organizational Culture"[Mesh]))) AND (("clinical education"[tiab] OR "clinical teaching"[tiab] OR "clinical supervision"[tiab] OR "clinical facilitation"[tiab] OR "clinical placement*"[tiab] OR "student placement*"[tiab] OR practicum[tiab] OR internship[tiab] OR "health professions education"[tiab] OR "nursing education"[tiab] OR "medical education"[tiab] OR "medical residenc*"[tiab] OR hospital*[tiab] OR "health service*"[tiab] OR "primary care"[tiab] OR "healthcare organization*"[tiab] OR "healthcare organisation*"[tiab] OR "Education, Professional"[Mesh] OR "Education, Nursing"[Mesh] OR "Education, Medical"[Mesh] OR "Hospitals"[Mesh] OR "Primary Health Care"[Mesh] OR "Internship and Residency"[Mesh])

Additionally, a manual search of the reference lists of all included studies will be conducted to identify potentially relevant publications that may not have been retrieved through the electronic search. Grey literature will also be searched to capture additional relevant sources that may provide valuable evidence but have not been published in peer-reviewed journals.

For Google Scholar, the search and record selection procedures will be explicitly described, including the screening limits applied and the relevance criteria used to identify eligible records. The dates of all searches, as well as any updates conducted prior to the final publication of the review, will be reported. The complete search strategies for all databases will be provided as supplementary material, in accordance with the methodological recommendations of the Joanna Briggs Institute and the PRISMA-ScR.

### Source of evidence selection

The review process will follow the structured sequence recommended by Peters and Marnie [20] in the JBI guidance: (1) All records retrieved from the database searches will be exported from a reference management software (EndNote) and shared in RIS format, including the complete set of references prior to the screening process. The records will then be imported into Rayyan, where duplicates will be removed and title and abstract screening will be performed. (2) The screening process will be conducted independently by two reviewers, who will assess the eligibility of studies according to the predefined inclusion and exclusion criteria. An initial pilot screening exercise will be undertaken to evaluate the clarity and applicability of these criteria. In cases of disagreement, the reviewers will discuss the study to reach consensus. If consensus cannot be achieved, a third reviewer will adjudicate the disagreement. Where necessary, the inclusion and exclusion criteria will be further refined to improve their clarity. (3) Once agreement has been achieved following the pilot exercise, title and abstract screening will be conducted for all retrieved records. (4) Studies meeting the eligibility criteria will then be retrieved in full text for detailed assessment. Any uncertainty regarding the eligibility of a full-text article will be resolved through discussion with the second reviewer. (5) Reasons for the exclusion of full-text articles will be documented in Rayyan as part of the review audit trail.

The electronic database searches will be updated immediately prior to completion of the review to identify recently published studies that may contribute relevant evidence. All data related to the study selection, screening, and data extraction processes will be made publicly available upon completion of the review to promote transparency and reproducibility.

For greater clarity and transparency, the study selection process will be presented in a flow diagram following the recommendations of the PRISMA 2020 Statement [21,22]. The flow diagram will document the number of records identified, screened, assessed for eligibility, and included in the review, as well as the reasons for exclusion at each stage of the selection process.

### Data extraction

Data will be extracted using a data extraction form developed by the review authors in a Microsoft Excel spreadsheet (Table 2). The extracted data will include demographic characteristics of the study population (e.g., age, nationality, and gender), methodological design, sample size, preceptors' professional discipline, type of preceptor training, and the extrinsic or contextual factors influencing preceptor performance (e.g., clinical, organizational, institutional, and social contexts). Additional data will include the health discipline of the students supervised by the preceptors and the level of education (undergraduate or postgraduate).

**Table 2:** Data extraction form.

ID
Reference
Methodological design
Sampling technique
Data analysis strategy
Population
Sample Size
Age
Gender
Country
Preceptors' professional discipline
Type of preceptor training
Extrinsic or contextual factors influencing preceptor performance
Health discipline of the supervised students
Level of education (undergraduate / postgraduate)

The data extraction tool will be iterative in nature and may be refined during the data extraction phase as new insights emerge from the analysis of the included sources. Any modifications made to the data extraction form will be documented and reported to ensure the transparency and reproducibility of the review process.

Data extraction will be conducted independently by each reviewer, who will serve both as a data extractor and as a verifier of the data extracted by a peer reviewer. Verification will be performed on a random sample comprising at least 50% of the studies assigned to each reviewer. Any discrepancies identified during the data extraction process will be resolved through consultation with a third reviewer. This procedure is intended to ensure the rigor, consistency, and reliability of the data collection process.

The authors of included studies may be contacted if relevant information required to address the research question is missing or unclear. The purpose of this contact is to ensure the most comprehensive data extraction possible by obtaining additional details that contribute to a more complete description and understanding of the phenomenon under investigation.

### Data analysis

The extracted data will be analyzed rigorously using both qualitative and quantitative approaches in three sequential phases.

(1) **Qualitative analysis:** The first phase will involve qualitative content analysis using an inductive approach [23,24]. The unit of analysis will be the contextual factors reported in each included study. Through an iterative process of reading, comparing, and grouping the data, thematic categories are expected to emerge, representing the clinical, organizational, institutional, and social factors that influence preceptor performance. The findings will subsequently be synthesized into a category map illustrating the emerging levels, categories, and hierarchical relationships among contextual factors. Differences across health disciplines and variations according to countries' income levels will also be explored.
(2) **Data triangulation:** The second phase will consist of investigator triangulation involving the entire review team. Each reviewer will independently read the extracted narratives at least three times to achieve an in-depth understanding of the data and to identify the essential features of the phenomenon under investigation [25,26]. Following this process, triangulation meetings will be held to compare and discuss the identified categories, reach consensus, and resolve any discrepancies in the interpretation and description of the findings.
(3) **Quantitative analysis:** The final phase will comprise two stages. First, descriptive statistics will be used to summarize the characteristics of the extracted variables (e.g., study design, sample size, participants' age and gender, country of origin, and other relevant study characteristics). Subsequently, a quantitative analysis of the emergent levels and categories of contextual factors will be conducted. This analysis will address the four review questions by describing the levels and categories of contextual factors and examining differences across health disciplines and according to countries' income levels. The findings from this phase will be presented in the form of tables, figures, and/or conceptual maps, as appropriate. A narrative summary will also be provided to describe how the findings address the objectives and research questions of the review.

### Quality assessment

It is important to note that the primary purpose of a scoping review is not to critically appraise the methodological quality of the included studies but rather to comprehensively map the existing evidence on the phenomenon under investigation. Accordingly, this review will focus on identifying and synthesizing the information necessary to address the research questions, including evidence derived from sources employing a wide range of methodological approaches and levels of methodological rigor. Although the value of critically appraising the quality of the evidence included is acknowledged, no formal methodological quality assessment will be undertaken, as this is consistent with the objectives of a scoping review. Instead, the emphasis will be placed on comprehensively mapping the contextual factors that influence preceptor performance across different health professions and countries with varying income levels, rather than evaluating intervention effectiveness, estimating effect sizes, or establishing causal relationships between variables, which are objectives more appropriately addressed through systematic reviews and meta-analyses.

Nevertheless, relevant methodological information from each included study will be reported, including study design, sample size and characteristics, data collection techniques, and data analysis strategies. Reporting these methodological characteristics will provide a broader understanding of the current state of research in this field while highlighting potential limitations associated with the methodological quality of the included studies.

### Protocol validation

The findings of this scoping review are expected to provide a comprehensive overview of the contextual factors that influence preceptor performance and the transfer of preceptorship competencies to clinical learning environments. Through a rigorous and systematic process of evidence identification and synthesis, the review aims to describe, classify, and enhance the understanding of the contextual factors that shape preceptor performance. It is anticipated that the review will identify the principal domains and themes reported in the literature, including factors related to the clinical, organizational, and institutional contexts. Differences in these contextual factors across health professions and according to countries' income levels will also be explored.

To strengthen the validity and methodological rigor of this protocol, the eligibility criteria, search strategy, and data extraction form were developed collaboratively by the review team and subsequently reviewed by a researcher with expertise in scoping review methodology. The preliminary search strategy was developed iteratively by progressively integrating each component of the Population–Concept–Context (PCC) framework and refining the search through repeated pilot testing. This process aimed to maximize the sensitivity and specificity of the search strategy to ensure the comprehensive identification of studies relevant to the review question.

Before the formal screening process, a pilot screening exercise will be conducted using a sample of records retrieved through the search strategy to assess the clarity, consistency, and applicability of the inclusion and exclusion criteria. Subsequently, two independent reviewers will screen titles, abstracts, and full-text articles and extract data using the predefined data extraction form. Any disagreements will be resolved through discussion and consensus, with consultation of a third reviewer when necessary. Any modifications to the methodological procedures made during the review process will be fully documented, justified, and reported in the final publication to ensure transparency and reproducibility.

Through these methodological procedures, this protocol is expected to provide a robust and reliable framework for synthesizing the existing evidence on the contextual factors that influence preceptor performance across diverse health professions and clinical education settings.

### Limitations

Given that the aim of this scoping review is to provide a comprehensive understanding of the contextual factors influencing clinical preceptor performance, and that a wide range of evidence sources have been included to achieve this objective, several limitations must be acknowledged.

The main limitation lies in the expected heterogeneity of the included studies, both in terms of study designs and methodological quality. In this regard, it should be noted that the review will include not only peer-reviewed journal articles but also grey literature. This heterogeneity may limit the comparability of findings and the development of generalizable conclusions.

With respect to publication bias, it is important to acknowledge that studies reporting non-significant findings may be more difficult to access, and that certain contextual barriers may remain underreported or not yet captured in the existing literature. Consequently, the findings of this review will provide a broad, but not exhaustive, overview of the phenomenon under study.

## Declaration of generative AI and AI-assisted technologies in the manuscript preparation process

During the preparation of this work, the authors used ChatGPT to assist with the translation of the manuscript from the authors' original Spanish text into academic English. The authors reviewed and edited the output as needed and take full responsibility for the content of the published article.

## CRediT author statement

**Bárbara Porter:** Conceptualization, methodology, data curation, writing original draft, resources, software, OSF registration. **Andrea Rodríguez:** Conceptualization, supervision, writing review and editing. **Ivonne Riquelme:** Conceptualization, validation, writing review and editing. **Maria José Montecinos:** Conceptualization, writing original draft, review and editing. **Sigrid Dömke:** Conceptualization, validation, writing review and editing.

## Funding

The present study received no external funding.

## Declaration of competing interest

The authors declare that they have no known competing financial interests or personal relationships that could have appeared to influence the work reported in this paper.

## Data Availability

No data was used for the research described in the article.
